# Predictability-Based Source Segregation and Sensory Deviance Detection in Auditory Aging

**DOI:** 10.3389/fnhum.2021.734231

**Published:** 2021-10-29

**Authors:** Christiane R. Neubert, Alexander P. Förstel, Stefan Debener, Alexandra Bendixen

**Affiliations:** ^1^Cognitive Systems Lab, Faculty of Natural Sciences, Institute of Physics, Chemnitz University of Technology, Chemnitz, Germany; ^2^Neuropsychology Lab, Department of Psychology, Carl von Ossietzky University of Oldenburg, Oldenburg, Germany

**Keywords:** auditory scene analysis, foreground-background separation, predictive coding, elderly listeners, temporal processing, Electroencephalography (EEG), mismatch negativity (MMN)

## Abstract

When multiple sound sources are present at the same time, auditory perception is often challenged with disentangling the resulting mixture and focusing attention on the target source. It has been repeatedly demonstrated that background (distractor) sound sources are easier to ignore when their spectrotemporal signature is predictable. Prior evidence suggests that this ability to exploit predictability for foreground-background segregation degrades with age. On a theoretical level, this has been related with an impairment in elderly adults’ capabilities to detect certain types of sensory deviance in unattended sound sequences. Yet the link between those two capacities, deviance detection and predictability-based sound source segregation, has not been empirically demonstrated. Here we report on a combined behavioral-EEG study investigating the ability of elderly listeners (60–75 years of age) to use predictability as a cue for sound source segregation, as well as their sensory deviance detection capacities. Listeners performed a detection task on a target stream that can only be solved when a concurrent distractor stream is successfully ignored. We contrast two conditions whose distractor streams differ in their predictability. The ability to benefit from predictability was operationalized as performance difference between the two conditions. Results show that elderly listeners can use predictability for sound source segregation at group level, yet with a high degree of inter-individual variation in this ability. In a further, passive-listening control condition, we measured correlates of deviance detection in the event-related brain potential (ERP) elicited by occasional deviations from the same spectrotemporal pattern as used for the predictable distractor sequence during the behavioral task. ERP results confirmed neural signatures of deviance detection in terms of mismatch negativity (MMN) at group level. Correlation analyses at single-subject level provide no evidence for the hypothesis that deviance detection ability (measured by MMN amplitude) is related to the ability to benefit from predictability for sound source segregation. These results are discussed in the frameworks of sensory deviance detection and predictive coding.

## Introduction

Hearing is a daily life challenge as the auditory system needs to disentangle the incoming sound mixture into meaningful streams by linking sounds belonging to one source together, and separating sounds belonging to different sources (Bregman, 1990). Sound segregation ability and the ability to track a particular sound source (e.g., a speaker) across time in a concurrent acoustic mixture is crucial to follow the target stream while ignoring background noise (e.g., other speakers, cafeteria noise). In most listeners, these auditory processes work surprisingly smoothly. The prevailing theoretical framework for explaining the ease with which listening in such complex environments works, is predictive coding (Friston, 2005; Winkler et al., 2009; Kanai et al., 2015). The core idea is that sound sources tend to behave regularly (predictably) over time, and once the brain has formed a predictive model for the emission pattern of a given sound source, this source can be tracked over time and separated from other sources without effort (Bendixen, 2014; Schröger et al., 2014; Winkler and Schröger, 2015), and even without attention (Sussman et al., 1999, 2007). As simple and elegant as this explanation seems, recent work has pointed toward some unresolved issues related to the predictive-coding account of auditory perception (Denham and Winkler, 2017; Heilbron and Chait, 2018). Both reviews reiterate the concern that detecting the predictability of a sound source does not imply forming actual predictions about this sound source, and that the underlying neural mechanisms are not entirely clear. Furthermore, the evidence for the brain’s capacity to detect the predictability of a sound source often comes from indirect measures, using the logic of occasionally violating the otherwise predictable pattern and measuring whether the brain responds to this violation (deviation) in a specific way (Schröger, 2007). Whether detecting a predictability violation (i.e., sensory deviance detection) and sensory prediction are indeed related, is notoriously difficult to demonstrate (Denham and Winkler, 2017; Heilbron and Chait, 2018).

In the current study, we set out to find a link between detecting predictability violations and using auditory predictability for sound source segregation. We addressed this question by exploiting inter-individual variability in those two capabilities, asking whether listeners whose auditory system detects deviants more readily (as evidenced by corresponding brain responses) can also use predictability more easily for segregating sound sources from one another (as evidenced by listening success in a challenging task with auditory background interference). We chose a sample of listeners aged 60–75 years, for two main reasons. First, we expect to find more inter-individual variability in this sample than in young individuals (Alain et al., 2006), which increases the statistical power for finding a relation between deviance detection and predictability-based source segregation if there is such a relation. Second, previous studies have pointed toward a need for explaining elderly listeners’ apparent deficits in complex sensory deviance detection (Getzmann and Näätänen, 2015; Rimmele et al., 2015) as well as in predictability-based source segregation (Rimmele et al., 2012a). Accordingly, providing evidence for such a relation would contribute to a better understanding of elderly listeners’ difficulties with complex acoustic scenes (Alain et al., 2006).

To study predictability-based source segregation, we capitalized on prior work showing that spectrotemporal regularities support auditory stream segregation (Bendixen et al., 2010; Andreou et al., 2011; Sohoglu and Chait, 2016; Aman et al., 2021). Specifically, it is easier to segregate interleaved auditory streams when one or all of them contain spectrotemporal regularities (patterns). This has been demonstrated when the stream carrying the regularities is relevant to the listeners’ task (Rimmele et al., 2012a; Aman et al., 2021) and also when the listener tries to ignore this stream (Andreou et al., 2011; Rimmele et al., 2012a). Evidence on whether this capacity to use regularities for stream segregation is preserved in elderly listeners is controversial: On the one hand, Rimmele et al. (2012a) suggest that elderly listeners can make use of predictability-based stream segregation when the stream carrying the regularity is task-relevant (see their Exp. 1), but not—at least not for all forms of regularity—when the regular stream is task-irrelevant and needs to be ignored (see their Exp. 2). Specifically, they found an age-related impairment in using an isochronous regularity in a background sound stream for ignoring this stream while performing a difficult foreground listening task. Rimmele et al. (2012a) interpret their findings in a predictive-coding framework by suggesting that spectrotemporal regularities stabilize auditory stream segregation and that the different levels of task relevance lead to different mechanisms of processing the regularities. On the other hand, de Kerangal et al. (2021) recently showed that the ability to track sources in an acoustic scene based on their regularities is largely preserved in elderly listeners. In their study, all streams were task-relevant, and the specific listening task was different from the one used by Rimmele et al. (2012a). The current study closely followed the task and design of Rimmele et al. (2012a) to assess whether elderly listeners’ impairment in using background regularities for stream segregation can be replicated.

Besides this replication attempt, a key aspect of the current study—as denoted above—was to relate each individual listener’s capability to use background regularities for stream segregation with their ability to extract such regularities. Regularity extraction was measured indirectly via the elicitation of specific brain responses by regularity violations. The key indicator was the mismatch negativity (MMN) component of the event-related brain potential (ERP) extracted from the participant’s electroencephalogram (EEG). The MMN is a component elicited by sensory events that violate some previously established regularity (Näätänen et al., 1978; for reviews, see e.g., Näätänen et al., 2007; Garrido et al., 2009; Fitzgerald and Todd, 2020). MMN can thus be used as an indirect indicator of regularity extraction (Schröger, 2005). It is elicited even without attention to the stimuli carrying the regularities and violations (e.g., Näätänen et al., 1993; Winkler et al., 2005). MMN is elicited by violations of simple rules (such as repetition of stimulus properties), but also of more abstract regularities such as certain patterns in which sounds are arranged (Zachau et al., 2005; for a review see Paavilainen, 2013). The MMN component is characterized by a frontocentral negativity with polarity inversion at the mastoids when using nose reference (Näätänen et al., 2001, 2005). Numerous studies have investigated MMN in elderly listeners, and many of them have found that its amplitude is attenuated and its peak latency is prolonged with aging (Alain and Woods, 1999; Cooper et al., 2006; Näätänen et al., 2011; Rimmele et al., 2012b; Cheng et al., 2013; Bartha-Doering et al., 2015; Getzmann and Näätänen, 2015).

To the best of our knowledge, no study has yet investigated whether MMN elicited by auditory regularity violations in elderly listeners shows a systematic relation with their ability to use regularities for stream segregation. We used the same spectrotemporal pattern as Rimmele et al. (2012a) to measure both auditory processes in the same listeners. We expected a significant correlation between MMN amplitude (as a proxy of auditory regularity extraction) and behavioral benefit from regular vs. random background sounds (as a proxy of regularity-based stream segregation). Finding such a correlation would strengthen the notion that extracting predictability and using predictability for decomposing acoustic mixtures are closely related processes, and would inform predictive-coding accounts of auditory perception.

## Materials and Methods

### Participants

30 volunteers aged 60–75 years participated in the study (17 female, 13 male; 29 right-handed, 1 left-handed; mean age 67.8 years, SD 4.1 years). All participants’ behavioral data were analyzed. Due to substantial artifacts in the EEG, two participants’ data were excluded from ERP data analysis (both participants were female; mean age of the remaining sample: 68.1 years, SD 4.1 years). The study was approved by the Ethics Committee of the University of Oldenburg. According to the Declaration of Helsinki, each participant gave written informed consent prior to the beginning of the experiment after all procedures had been explained. Participants received a modest financial compensation (8 €/h) for their participation.

### Experimental Stimuli and Apparatus

Sounds were created with Matlab (R2012b) and the stimulus delivery was controlled using the Psychophysics Toolbox extension for Matlab (Psychtoolbox 3.0.10). Instructions, visual cues during the training phase and the movie were presented on a wall-mounted TFT monitor. A Soundblaster X-Fi Audio interface was used to generate the audio signals. It was connected to a Tucker-Davis attenuator in bypass mode, which in turn was connected to a Denon PMA 510AE amplifier. Sounds were delivered via a pair of Cambridge Audio S30 speakers, positioned approximately 1.5 m away from the participant on both sides of the TFT monitor in the experimental room. Participants sat comfortably inside an electrically and acoustically shielded chamber while performing the experimental tasks.

#### Stream Segregation Part (Active Task)

**FIGURE 1:**
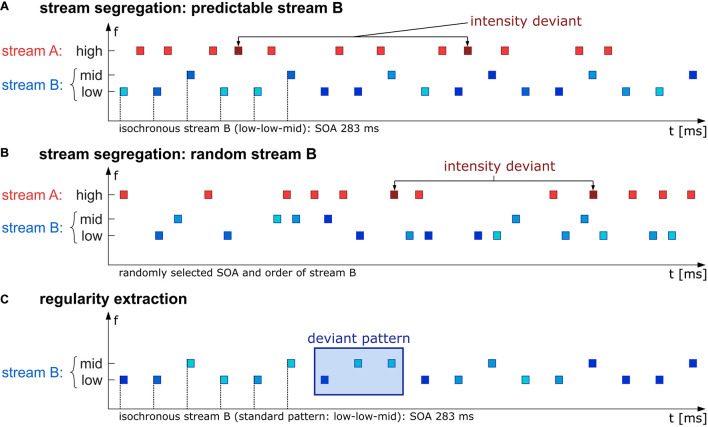
Schematic illustration of the stimulus paradigm. In the first experimental part, participants were instructed to detect intensity deviants (probability in sequence 10%, deviants are 10 dB higher in level than standards) in a high-frequency stream A (red rectangles). The task-irrelevant stream B (blue rectangles) was presented in either an isochronous (SOA 283 ms) and therefore predictable low-low-mid pattern **(A)** or random in the chosen SOA (160, 270, or 420 ms) and frequency (either low or mid, **B**). The second experimental part was a passive listening condition in which just the B stream was presented (isochronous, SOA 283 ms) containing standard low-low-mid triplets which were rarely interrupted by a deviant low-mid-mid triplet with probability of 17% **(C)**. SPL levels in stream B across all conditions **(A–C)** varied between 55 and 75 dB illustrated by different blue hues.

Following Rimmele et al. (2012a), the behavioral task was set up such that two auditory streams were interleaved and that listeners had to perform a task (intensity deviant detection) in one of them (the “A” stream), while the other one (the “B” stream) interfered with the task. This interference was caused by random intensity variation in the “B” stream, which obscured the intensity regularity in the “A” stream and thus impeded deviance detection as long as tones from the “A” and “B” stream were perceptually integrated. Accurate task performance thus required perceptual segregation of streams A and B; in turn, task performance gives an indirect measure of stream segregation (see Micheyl and Oxenham, 2010; Andreou et al., 2011; Rimmele et al., 2012a; for the same measurement logic). Specifically, the stimulus set consisted of short sinusoidal tones with a duration of 60 ms, including 5 ms half-raised cosine on- and offset ramps. The stimuli had three different frequencies: 370 Hz (“low”), 440 Hz (“mid”), and 554 Hz (“high”), presented in rapid succession such that they can be interpreted as concurring streams “A” (high tones) and “B” (low and mid tones, see Figure 1). The task-relevant stream A was presented with a level of 60 dB(C-weighted) for standards, while the level of rare intensity deviants (10% of the stimuli in stream A) was increased by 10 dB. Deviants were randomly placed with the restriction of 1,500 ms minimum distance between any two deviants. The stimulus onset of the tones in stream A was pseudo-randomized and therefore unpredictable. The stimuli in stream A were presented with 80% occurrence probability uniformly distributed between any two tones of stream B, always leaving at least 15 ms silence between all tones to avoid simultaneous presentation. The task-irrelevant stream B consisted of mid and low tones, whose spectrotemporal predictability varied with the experimental condition. Level of tones in stream B varied randomly in both conditions (55–75 dB(C) in 1 dB steps). This value range was chosen to interfere with the deviant detection task as soon as stream segregation would fail. In the predictable condition, stream B followed an isochronous low-low-mid order with a constant SOA of 283 ms. In the random condition, stimuli in stream B were not presented in a spectrotemporal pattern; instead, the SOA was randomly chosen from three discrete values (160, 270, or 420 ms), and tone frequencies were randomly chosen from the different frequency values (370 or 440 Hz). Mean SOA was equal to the predictable condition, and the proportion of different frequency values was also kept identical to the predictable condition (i.e., twice as many low tones as mid tones).

In both conditions, participants were instructed to indicate intensity deviants (targets) in stream A with a mouse click. To control for laterality effects, half of the participants answered with the index finger of the right hand, the other half answered with the index finger of the left hand. The total number of deviant tones per condition (predictable or random) was 255, distributed to three blocks per condition. Each block had a duration of approximately 5 min. In the six blocks, the two conditions were presented in an alternating scheme, with the starting condition counterbalanced across participants.

#### Regularity Extraction Part (Passive-Listening Task)

In the regularity extraction part, only stream B (low and mid tones) of the predictable condition from the stream segregation part was presented. Instead of the standard low-low-mid triplet, a rule-breaking low-mid-mid triplet was interspersed with 17% probability. These deviant triplets were expected to elicit an MMN. During the passive-listening part, participants watched an emotionally neutral excerpt of a muted documentary. The measurement lasted 15 min, without breaks, including 180 deviant low-mid-mid triplets.

### Procedure

Before the main experiment started, participants completed a four-level training procedure with increasing difficulty. In level 1, only the task-relevant stream A was presented, and visual support was given (a white square indicating the occurrence of a deviant tone). In level 2, stream A was presented alone without visual aid. In level 3, both streams (A and B) were presented with visual support for the deviant tone in stream A. In level 4, both streams were presented without visual aid; the procedure was thus identical to the experimental blocks. The training blocks lasted 1 min each and could be repeated if necessary (level 4 was always repeated at least once). Training was finished when performance reached a stable level and the participant had notably understood the task. After EEG preparation, one additional training block (level 4) was presented to refresh the knowledge of the task.

The main experiment consisted of two parts with EEG recordings throughout. First, participants completed the behavioral experiment (stream segregation part). Second, they were presented with the stimuli of the regularity extraction part while watching the silent documentary. After removing the EEG cap, the pure-tone audiogram (*via* Siemens Unity II audiometer and Sennheiser HAD-200 headphones) was measured at octave frequencies between 125 Hz and 8 kHz for both ears. To measure speech-in-noise comprehension, the Oldenburg Sentence Test (OLSA,^1^ Wagener et al., 1999b) was administered, using the adaptive procedure at a noise level of 65 dB SPL (presented with calibrated Siemens CD 310 F free field speakers). In addition, participants filled questionnaires on demographic variables, and they completed the Mehrfachwahl-Wortschatz-Intelligenztest (MWT-B, Lehrl, 1977) as a short screening for verbal intelligence.

The whole experimental session lasted between 2.5 and 3.5 h, including all tests and tasks, electrode application and removal as well as breaks for the participants.

### Electroencephalogram Recording

EEG data were continuously recorded using a BrainAmp amplifier system (BrainProducts, Gilching, Germany) with passive Ag/AgCl electrodes from 96 scalp positions using an infracerebral electrode cap with an equidistant electrode layout (Easycap, Herrsching, Germany). The horizontal electrooculogram (EOG) was measured with electrodes placed at the outer canthi of the left and right eye. The vertical EOG was obtained from separate electrodes placed below the left and right eye and from two electrodes above the eyes that were inserted in the electrode cap. The reference electrode was placed at the tip of the nose. EEG and EOG signals were amplified and recorded with a sampling rate of 500 Hz, applying an analog filter with 250 Hz low pass and 0.0159 Hz high pass (time constant 10 s).

### Data Analysis

#### Hearing Tests

**FIGURE 2:**
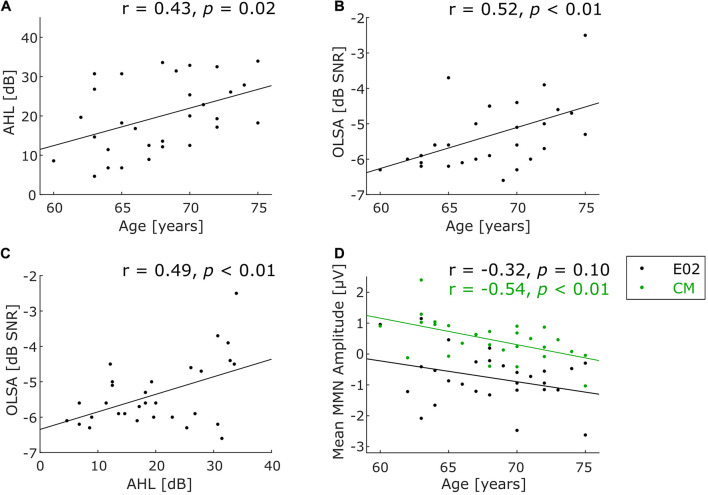
Scatterplots for characterizing the participant sample. **(A)** Correlation of age and peripheral hearing status, **(B)** correlation of age and speech-in-noise comprehension, **(C)** correlation of the hearing tests with one another, **(D)** correlation of age and deviance detection measured by MMN at frontocentral electrode position (E02, black dots) and common mastoids (CM, green dots). Note that only 28 participants were included for the latter correlation (see text for details). Significantly positive correlations indicate that higher age is associated with higher average hearing loss (**A**, *p* = 0.02) and with worse speech-in-noise comprehension (**B**, *p* < 0.01). Moreover, higher average hearing loss is associated with worse speech-in-noise comprehension (**C**, *p* < 0.01). A significant negative correlation between MMN amplitude and age only for CM shows less positive amplitudes with increasing age (**D**, green line, *p* < 0.01).

To calculate an aggregate measure for the peripheral hearing status, the average of the measured thresholds in the audiogram from 0.125 to 8 kHz across both ears was calculated (average hearing loss, AHL). To measure speech comprehension, the OLSA result yields the signal-to-noise ratio at which 50% of the speech material is still understood (50% speech recognition threshold in dB SNR). Pearson correlation coefficients were calculated for correlations between age and AHL (Figure 2A), age and OLSA (Figure 2B), as well as AHL and OLSA (Figure 2C).

#### Behavioral Data

During the stream segregation part, participants’ responses and response times were recorded. Using signal detection theory, the sensitivity index d’ was calculated separately for the two conditions (predictable, random). The d’ calculation was adapted to account for the rapid stimulus presentation (Bendixen and Andersen, 2013). Specifically, if two consecutive button presses occurred within less than 50 ms from one another, the second one was marked as an accidental key press, and only the first one was counted for the analysis. All responses that occurred within 0.1–1.2 s after a target (intensity deviant onset) were counted as hits. Note that response windows between two targets never overlapped due to the minimal distance between two deviant stimuli of 1.5 s. All remaining button presses (i.e., those that were not counted as hits or accidental presses) were classified as false alarms. The proportion of hits was calculated by dividing the number of hits by the number of targets. The proportion of false alarms was adapted to the rapid stimulus presentation in the following way (Bendixen and Andersen, 2013): Conceptually, the experimental block duration was separated into response windows of 1.1 s duration (the defined response window for targets), and the number of false alarms was divided by the number of such windows in which false alarms could occur (i.e., without response windows for targets; for detailed methods see Bendixen and Andersen, 2013). Afterward the sensitivity index was calculated [d’ = z(pHits)—z(pFA)] with z transformation by the inverse of the normal cumulative distribution function. To solve the problem that 100 or 0% hits or false alarms would result in plus or minus infinity, all proportion values were transformed by adding 0.5 to the individual hit and false alarm numbers and dividing the resulting score by the number of target or false alarm intervals adding one interval (Hautus, 1995). Due to this transformation and the finite number of targets and non-target intervals, a maximum d’ sensitivity score of 6.01 would be achieved with perfect performance in each condition.

**FIGURE 3:**
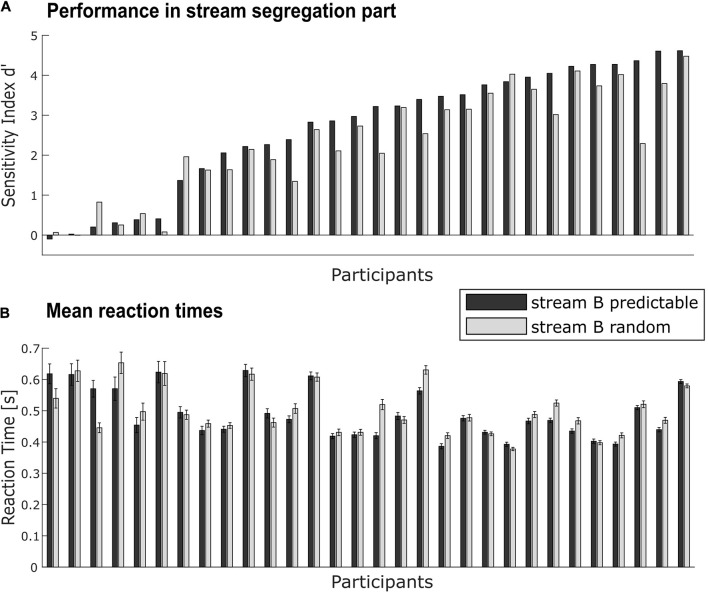
Individual performance in the target detection task. **(A)** Performance for each participant (*N* = 30) as measured by sensitivity d’ for predictable (black bars) and random conditions (gray bars). **(B)** Mean reaction times to detected targets in the task-relevant stream for each participant in both conditions (notation as in **A**). Error bars indicate the standard error in each condition within the participant. Data in both panels is sorted by performance in the predictable condition.

Sensitivity indices d’ were statistically analyzed by two-tailed *t*-tests against zero separately for each conditions (predictable, random, see Figure 3A). They were compared between the two conditions with a paired-sample two-tailed *t*-test. To quantify a possible advantage (i.e., higher d’ score) in the predictable relative to the random condition, the benefit Δd’ was calculated, subtracting d’ of the random condition from d’ of the predictable condition. To analyze correlations between benefit and possible contributing factors like age, regularity extraction ability (measured by MMN), peripheral hearing status (AHL), and speech-in-noise comprehension (OLSA), Pearson correlation coefficients were calculated.

Reaction times were calculated separately for each condition in the stream segregation part (Figure 3B) and compared against each other with a paired-sample two-tailed *t*-test.

#### Electroencephalogram Pre-processing

Data analysis was carried out with Matlab R2020b (The MathWorks Inc., Natick, United States) and the toolbox EEGLAB (Delorme and Makeig, 2004) version 14.1.1b. EEG data were decomposed into independent components (independent component analysis, ICA) with the extended Infomax algorithm (Bell and Sejnowski, 1995). Prior to and only for the purpose of ICA, data were high-pass filtered with a Kaiser-windowed sinc finite impulse response (FIR) filter (cutoff frequency: 1 Hz, filter order: 9056, Kaiser β: 5.65326, transition bandwidth: 0.2 Hz, maximal passband ripple: −60 dB), and artificial consecutive epochs of 1 s length containing non-stereotypical artifacts (as identified by eeglab’s rejkurt and jointprob functions with thresholds of 3 STD) were rejected. The independent components were saved in an untreated dataset (i.e., without the 1-Hz filter and the epoch rejection), and artifact-related component activity comprising eye movements, eye blinks, cardiac signals, muscle noise, and line noise were identified according to independent judgments by two of the authors (CN, AB). Subsequently, EEG data were filtered with a 0.1–30 Hz bandpass FIR filter (Kaiser-windowed, filter order: 9056, Kaiser β: 5.65326, transition bandwidth 0.2 Hz, passband ripple: −60 dB). In some participants, few channels (maximum 3) with high amounts of residual artifact were replaced by using spherical interpolation. Epochs of 950 ms duration, including a 100 ms pre-stimulus interval used for baseline correction, were extracted from −100 to 850 ms relative to stimulus onset of the second stimulus in the low-low-mid standard triplet or the second tone in the low-mid-mid deviant triplet. With 850 ms post-stimulus, the epochs comprised exactly three tones. Epochs with amplitude changes exceeding 100 μV on any channel were rejected from further analysis. This left two participants with less than 70% artifact-free epochs; the corresponding datasets were excluded from further ERP analysis (see above). The remaining datasets showed an average data loss of 7.0% in the passive listening condition, with 621–878 remaining epochs per participant (Mean = 817, *SD* = 59) for standard triplets, and 129–180 remaining epochs (Mean = 167, *SD* = 13) for deviant triplets.

#### Event-Related Brain Potentials

**FIGURE 4:**
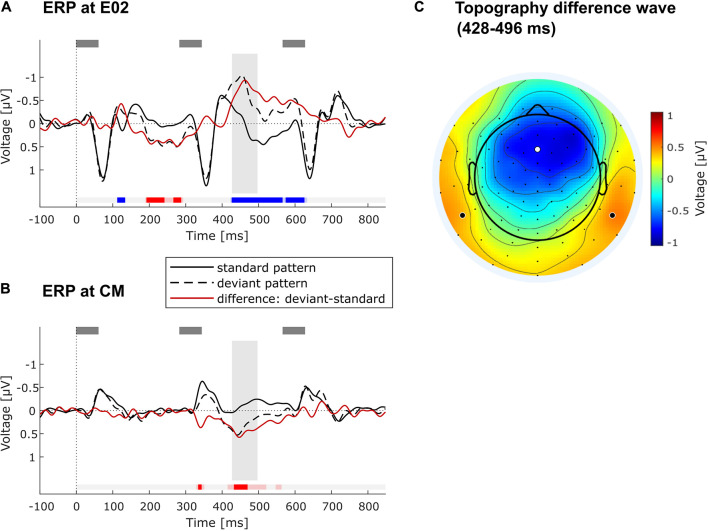
ERP results. **(A)** Grand-average ERPs at frontocentral electrode position E02 across all included participants (*N* = 28) elicited by standard (solid black line) and deviant triplets (dashed black line) as well as their difference wave (red line). Gray rectangles above denote the tones. Timepoint 0 refers to the onset of the second tone in low-low-mid (standard) or low-mid-mid triplet (deviant). Blue/red markings under the ERPs indicate significant negative/positive deviation of the difference wave from zero as determined in running *t*-test with FDR-correction of the alpha level. Faint blue/red markings show significant negative/positive deviation with alpha level *p* < 0.05 (without correction). The gray vertical rectangle highlights the chosen time window (428–496 ms) around the peak of the negativity in the difference wave. **(B)** Grand-average ERPs at common mastoids (CM) across all included participants (*N* = 28). All markings, rectangles and lines have the same meaning as in **(A)**. **(C)** Topography of the difference wave in the chosen time window (428–496 ms), showing a frontocentral negativity with polarity inversion at the mastoids. The white dot indicates the location of E02 used for the ERP plot in **(A)**. Bold black dots show channel locations of left and right mastoid (both were averaged for ERP plot in **B**).

Data from the remaining 28 participants were used to form grand-average ERPs per stimulus type (standard or deviant triplet) in the regularity extraction part of the experiment. Difference waves were calculated by subtracting the average ERP for each participant elicited by standard stimuli from that elicited by deviant stimuli. The difference wave of the grand-average ERP was examined at the frontocentral electrode E02, which is located on the midline between Fz and FCz (Figure 4A), and at the average of the mastoids (common mastoids, CM, Figure 4B). These are typical locations to quantify the auditory MMN component (Näätänen et al., 2007). The difference wave was tested for statistically significant deviations from zero by means of a sample-wise running *t*-test throughout the whole epoch window (i.e., from 0 to 850 ms), correcting for multiple comparisons via the false discovery rate (FDR, Benjamini and Hochberg, 1995). After confirming a significant frontocentral negativity with this procedure, the negativity was further characterized by using the average voltage in a latency range from 428 to 496 ms after the onset of the second tone in the triplet. This latency range was chosen to start with the first sample of the longest number of consecutively significant samples at E02 in the FDR-thresholded running *t*-test (428 ms), to cover the frontocentral peak in the grand-average difference wave (462 ms), and to extend symmetrically to the other side of the peak (496 ms). It should be noted that peak-picking procedures are being criticized for their circularity (“double dipping”) (Kriegeskorte et al., 2009; Luck and Gaspelin, 2017). In the current case, we chose the latency range (428–496 ms) *after* having determined that a significant negativity was elicited for every data sample in that range, and we used it for individual MMN amplitude quantification and for studying the scalp topography (Figure 4C).

## Results

### Hearing Status and Verbal Intelligence

Average hearing loss (AHL) values across all frequencies ranged from 4.64 to 33.93 dB (Mean = 19.88 dB, *SD* = 9.14 dB), indicating wide variance from participants with almost normal thresholds to participants with considerable peripheral hearing loss. A significant correlation between AHL and age was observed (*r* = 0.43, *p* = 0.02, *N* = 30; see Figure 2A), indicating that peripheral hearing ability decreased with increasing age within our sample spanning 15 years of age. To exclude a confounding effect of hearing status on task performance, AHL in the frequency range in which the experimental stimuli were presented (370–554 Hz) was specifically examined. As a proxy, AHL values for 250 and 500 Hz were averaged, yielding a mean AHL of 5.42 dB (*SD* = 6.83 dB, range −7.5 to 20 dB). This indicates relatively preserved hearing at the frequencies of the auditory stimuli.

OLSA results ranged from −6.6 to −2.5 dB SNR (Mean = −5.36 dB SNR, *SD* = 0.93 dB SNR). This suggests a mild to moderate impairment in understanding speech in noise relative to the expected average threshold of young normal-hearing subjects at −7.1 dB SNR (Wagener et al., 1999a). Note that numerically lower—more negative—values indicate better performance. Speech-in-noise thresholds were significantly worse (i.e., less negative) with increasing age (*r* = 0.52, *p* < 0.01, *N* = 30; Figure 2B). Moreover, AHL correlated significantly with speech-in-noise comprehension (*r* = 0.49, *p* < 0.01, *N* = 30; Figure 2C).

All participants scored at or well above the population average on the verbal intelligence test MWT-B (verbal IQ range 97–145, mean IQ: 127), which rules out occurrences of major cognitive decline in the sample.

### Behavioral Data: Performance and Predictability-Based Performance Benefit

Performance as evaluated by the sensitivity index d’ scores ranged from −0.10 to 4.62 in the predictable condition (Mean = 2.69, *SD* = 1.52) and from 0 to 4.48 in the random condition (Mean = 2.35, *SD* = 1.33). The variation across participants was large (see Figure 3A), with some participants performing very well (note that performance without misses and false alarms would lead to a d’ maximum of 6.01) and other participants with d’ near zero failing to handle the target detection task above chance level. On average, performance in both conditions was significantly above chance [predictable condition: *t*(29) = 9.69, *p* < 0.001; random condition: *t*(29) = 9.66, *p* < 0.001].

Performance in the predictable condition was significantly better than in the random condition, *t*(29) = 3.39, *p* < 0.01. This implies that at group level, participants can benefit from the spectrotemporal regularity in the task-irrelevant background for performing the foreground task. At single-subject level, not all participants showed the same amount of benefit (or a benefit at all): Δd’ values ranged from −0.62 to 2.08 (Mean = 0.34, *SD* = 0.54).

Mean reaction times (see Figure 3B) to detected targets in the task-relevant stream did not differ between conditions [*t*(29) = −1.25, *p* = 0.22; predictable condition: Mean = 0.49 s, *SD* = 0.08 s; random condition: Mean = 0.50 s, *SD* = 0.08 s].

### Event-Related Brain Potential Data: Deviance Detection

FDR-corrected running *t*-tests of the difference wave (deviant minus standard) at the frontocentral electrode position E02 show significant negative and positive deflections in several time ranges, of which the longest one was taken for further statistical analysis (see Figure 4A). It consists in a pronounced negativity in a late time range, starting from 428 ms after the onset of pattern-violating second tone (low-*mid*-mid instead of low-low-mid). The topography of this late component (measured from 428 to 496 ms) shows a frontocentral negativity with inversed polarity at the mastoids (Figures 4B,C), which is consistent with a generator of this component in auditory cortex (Näätänen et al., 2007). The negativity was identified as an MMN (see below for discussion). Individual MMN amplitudes at the frontocentral electrode location E02 in the time window 428–496 ms varied widely from −2.62 to 1.15 μV (Mean = −0.77 μV, *SD* = 0.87 μV; mean amplitude was significantly negative at group level: *t*(27) = −4.65, *p* < 0.001). No significant correlation between frontocentral MMN amplitude and age was found (*r* = −0.32, *p* = 0.10, *N* = 28, black dots in Figure 2D). Individual MMN amplitudes at common mastoids (CM) varied in the chosen time window from −1.03 to 2.40 μV (Mean = 0.47 μV, *SD* = 0.66 μV; mean amplitude was significantly positive at group level: *t*(27) = 3.78, *p* < 0.001). MMN amplitudes at common mastoids were found to significantly correlate with age (*r* = −0.54, *p* < 0.01, *N* = 28, green dots in Figure 2D), with less positive MMN amplitudes associated with increasing age. The correlations between MMN amplitude and age at E02 and CM do not qualitatively change when controlling for AHL.

### Correlations Between Predictability-Based Performance Benefit, Deviance Detection, and Auxiliary Data

**FIGURE 5:**
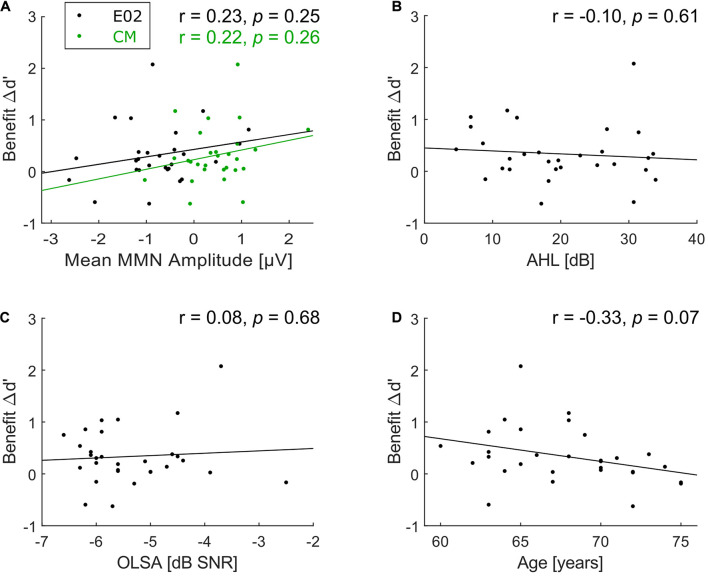
Scatterplots of correlations with benefit. **(A)** Correlation between benefit Δd’ and mean MMN amplitude at frontocentral electrode position E02 (black dots) and common mastoids (CM, green dots; note that just 28 participants were included for both correlations), **(B)** between benefit Δd’ and average hearing loss (AHL), **(C)** between benefit Δd’ and speech-in-noise comprehension, and **(D)** between benefit Δd’ and age. No significant correlations with benefit Δd’ were found.

Correlation analyses were carried out in search of underlying factors for the wide variation in the predictability-based performance benefit (Δd’). No significant correlation was observed between Δd’ (as a measure of predictability-based performance benefit) and MMN amplitude (as a measure of deviance detection capacities) at E02, *r* = 0.23, *p* = 0.25, *N* = 28 (Figure 5A, black dots) and CM, *r* = 0.22, *p* = 0.26, *N* = 28 (Figure 5A, green dots). There was also no correlation between benefit and AHL (*r* = −0.10, *p* = 0.61, *N* = 30, Figure 5B) nor between benefit and speech-in-noise comprehension (*r* = 0.08, *p* = 0.68, *N* = 30, Figure 5C).

**FIGURE 6:**
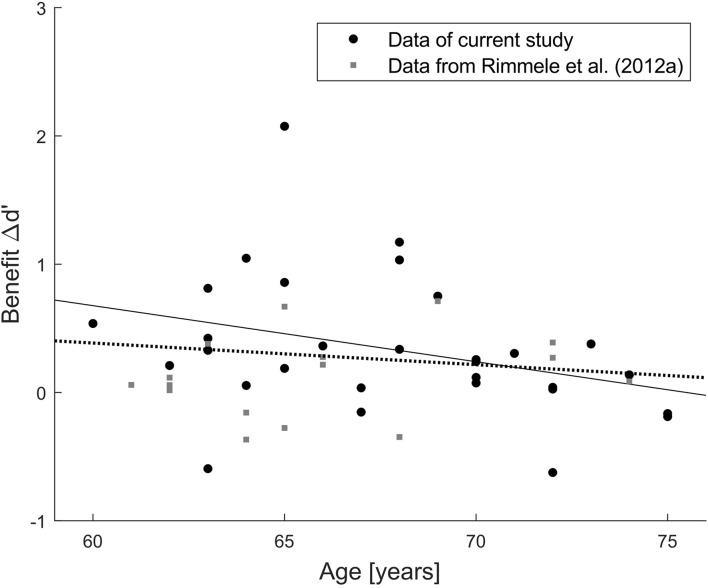
*Post hoc* joint analysis of two independent participant samples. Scatterplot of the relation between age and benefit Δd’ for performance in the predictable/isochronous minus random condition. Each dot refers to one individual data point (black dots: current study, gray squares: Exp. 2 in Rimmele et al., 2012a). Solid line indicates correlation in the current study (*r* = –0.33, *p* = 0.07, *N* = 30), dotted line indicates correlation in the combined data (*r* = –0.14, *p* = 0.34, *N* = 46).

Examining the correlation of benefit and age suggests that there could be a trend toward lower benefit with increasing age (*r* = −0.33, *p* = 0.07; Figure 5D), though it does not meet the conventional alpha level of 5% (and much less a Bonferroni-corrected alpha level of 1% to compensate for the five correlation coefficients computed for benefit). This numerical association of benefit and age could reflect a spurious trend, or it could indicate a real effect measured with too low power given the relatively small sample size (*N* = 30). To separate between these two possibilities, we retrieved the data of all 16 participants (mean age = 65.9 years, *SD* = 4.0 years) from the elderly group of Experiment 2 by Rimmele et al. (2012a). The current experimental design is highly similar to Experiment 2 of Rimmele et al. (2012a), thus their data were re-analyzed in terms of Δd’ benefit as in the current study by subtracting d’ in the random condition from d’ in their isochronous condition (corresponding to the predictable condition here). A joint correlation analysis based on data from both studies (Figure 6) revealed no significant correlation between age and benefit (*r* = −0.14, *p* = 0.34, *N* = 46), which suggests that the trend in the current experimental data was indeed spurious.

### Further Control Analyses

Importantly, Rimmele et al. (2012a) had found no significant benefit for elderly participants in the isochronous condition (Mean d’ = 2.55, *SD* = 0.70) relative to the random condition (Mean d’ = 2.41, *SD* = 0.74). In order to identify reasons for the apparent discrepancy with the current result, a *post hoc* independent-samples *t*-test was performed to compare the Δd’ results from Rimmele et al. (2012a) with the current data. Δd’ was numerically higher in the current study (Mean = 0.34, *SD* = 0.54) than in the previous study (Mean = 0.13, *SD* = 0.32), but this difference was not statistically significant [*t*(44) = 1.38, *p* = 0.17]. Again, a joint analysis of both datasets showed that Δd’ is significantly different from zero across both groups of elderly participants [Mean = 0.27, *SD* = 0.48, *t*(45) = 3.71, *p* < 0.001].

The lack of significant correlation of behavioral benefit (Δd’) with any other measured variable (Figure 5) led us to examine the robustness of Δd’ measurement, to rule out that measurement error underlies the absence of correlation. We assessed the robustness of the measurement by calculating Δd’ separately for each consecutive pair of blocks (i.e., subtracting d’ in the first random block from d’ in the first predictable block, etc.)^2^ and determining Cronbach’s alpha of the three Δd’ estimates. Across our 30 listeners, Cronbach’s alpha was modest (0.31). Examination of the individual data showed that one single listener exhibited excessive variation between blocks^3^ whereas all others’ data were much more consistent. Excluding this one listener’s data increased Cronbach’s alpha to a moderate level of 0.58. Given that Δd’ is a difference score, 0.58 is an acceptable value, and it is considerably higher than the observed correlation of Δd’ with any other measured variable.

## Discussion

The current study was designed to measure elderly listeners’ abilities to extract an auditory spectrotemporal regularity (as evidenced by MMN responses to regularity violations) and to use the same regularity for stream segregation (as evidenced by enhanced listening performance in a foreground stream when the regularity is embedded in the to-be-ignored background). We expected to find a correlation between MMN amplitude (regularity extraction) and behavioral benefit (regularity-based stream segregation). In contrast to our hypothesis, we did not observe such a correlation, although both abilities were clearly present at group level and inter-individual variability was substantial in both of them. The two abilities and the absence of their association are discussed in turn below.

### Pattern Regularity Extraction in Elderly Listeners

To study whether elderly listeners can extract spectrotemporal patterns, we used MMN as an indirect measure of regularity extraction (Schröger, 2005). The “low-mid-mid” deviations from the “low-low-mid” pattern indeed elicited a significant ERP component whose topography is consistent with the auditory MMN component (Näätänen et al., 2001, 2005). The time-range of the MMN component was relatively late, with its peak at 462 ms relative to the onset of the deviant event (second tone in the triplet). MMN usually occurs at about 100–250 ms after deviation onset (Näätänen et al., 2007; Garrido et al., 2009). One possible reason for the late MMN is that the three stimuli of the tone pattern are perceptually bound into an auditory object and that the comparison of the sensory input with the expected template (whose mismatch leads to MMN) takes place after the last tone of the triplet. Relative to this third tone, the observed peak latency is 179 ms, which is well in line with the usual MMN latency (Näätänen et al., 2007; Garrido et al., 2009). Alternatively, an embedded regularity may be encoded in different ways (Horváth et al., 2001), not only in the form of a global pattern regularity (“low-low-mid is continuously repeated”) but also in the form of a local transitional regularity (“a mid-tone is always followed by a low tone”). This transitional regularity is not violated until the third tone of the triplet, which explains the occurrence of a late MMN as well. Other alternatives are that the late negativity reflects the typical prolongation of MMN latency in elderly listeners (Cooper et al., 2006; Näätänen et al., 2011; Getzmann and Näätänen, 2015), or that this negativity is not the typical MMN but the late discriminative negativity (LDN) following MMN, which has been described mainly for children (e.g., Cheour et al., 2001), but also for adults when abstract regularities are employed (Zachau et al., 2005; Bendixen et al., 2012). Altogether, we conclude that the negativity peaking at 462 ms reflects an automatic violation detection process, in which the deviant triplet violates the formed prediction about the sensory input and the predictive model needs to be updated (Winkler and Czigler, 1998).

One may object that comparing ERPs to standard (low-low-mid) and deviant triplets (low-mid-mid) is not ideal due to the physical difference in the second tone (low vs. mid tone). In fact, a significant early difference between standard and deviant ERP traces emerged in the latency range of the N1 component at about 120 ms after onset of the physically different tone (see Figure 4A). To differentiate whether this N1 enhancement for the deviant triplet was due to the physical difference or reflects an early sign of “true” deviance detection (Näätänen et al., 2005), it would have been more advantageous to swap the role of deviant and standard triplets half-way during the passive listening condition. Yet although this has been a standard recommendation in auditory MMN research for many years, recent work on the so-called “primacy bias” (Todd et al., 2011, 2013, 2014; Fitzgerald and Todd, 2018) has established that swapping standard and deviant comes with significant reductions in the overall size of the MMN component. This is because a “lasting first impression” of stimulus probabilities and significance has been formed, reducing the MMN elicited by the deviant that was first experienced as a standard (Todd et al., 2011, 2013, 2014; Fitzgerald and Todd, 2018). Such higher-order effects (signifying predictive coding at different timescales) would reduce overall MMN amplitude and might introduce another source of inter-individual variability. This might have interacted with the aim to quantify pattern MMN on an individual basis in the current study. Importantly, even if the early (120 ms) negativity elicited by deviant relative to standard triplets was confounded by physical difference, it is highly unlikely that this translated to the negativity at 462 ms analyzed here, because the third tone in standard and deviant triplets was physically identical and the early modulation was much smaller than the late MMN.

To sum up, the ERP data from passive listening suggest that—at group level—elderly listeners extracted the pattern regularity and detected deviations from it. Finding this ability at group level is consistent with prior work on pattern regularity extraction in elderly listeners (Alain and Woods, 1999; Näätänen et al., 2011; Rimmele et al., 2012b; Getzmann and Näätänen, 2015). The respective abilities of the individual listeners (as quantified by MMN amplitude) showed a high amount of variation, which was later used to address the question of a possible direct relation with the ability of regularity-based stream segregation.

Note that evidence in favor of pattern regularity extraction in elderly listeners does not imply that this ability is *fully* preserved in elderly listeners: this conclusion would require comparison with a young-listener control group. Such a control group was not included in the current study because our focus was on examining differences *within* a group of elderly listeners (60–75 years), not on drawing comparisons across wide age ranges (∼25 years vs. ∼65 years, as in many other studies). Previous studies comparing widely different age groups have consistently found smaller frontocentral MMN amplitudes in elderly as compared to young listeners (Alain and Woods, 1999; Näätänen et al., 2011; Rimmele et al., 2012b; Getzmann and Näätänen, 2015). The fact that we did not find a correlation between frontocentral MMN amplitude and age in the current study (Figure 2D, black dots) does not contradict those prior observations, as one would expect the chronological age to play a more minor role in a sample spanning 15 years (60–75 years) than in samples spanning 40 years of age and more.

Indeed, the correlation between polarity-reversed MMN amplitude at the common mastoids and age was significant in our sample (Figure 2D, green dots). This might indicate that elderly listeners show impairments in the supratemporal but not frontal MMN generators for deviations of our pattern regularity (Giard et al., 1990; Opitz et al., 2002; Näätänen et al., 2007), or it may reflect a shift in the orientation of the superior temporal gyrus dipole (Deouell, 2007). Since our frontocentral MMN amplitude numerically shows an almost parallel trajectory with age as the mastoid MMN amplitude (Figure 2D: both tend to become less positive/more negative with age), the latter explanation seems more likely (see Baldeweg et al., 1999, for a similar observation where frontal MMN increase is paralleled by mastoid MMN decrease, though not in the context of aging). More specifically, it seems plausible that a change in orientation of the superior temporal gyrus dipole reduces MMN amplitudes at mastoid electrodes and increases MMN amplitudes at frontocentral electrodes at the same time. In contrast, a change in strength of the superior temporal gyrus dipole would reduce MMN amplitude at both electrode sites, and one would have to assume a parallel increase in strength of the frontal MMN source to account for the observation that MMN decreases with age at the mastoids while it tends to increase with age at frontocentral electrodes. While this would be a less parsimonious explanation, the current data should not be over-interpreted regarding this issue, especially since the association of age and frontocentral MMN amplitude was not significant by conventional criteria. It is likewise difficult to relate this observation to other MMN studies investigating aging effects by comparing young and elderly listeners, because many studies report frontocentral MMN amplitudes that are directly referenced to the mastoid electrodes, or do not report mastoid data at all. Further studies examining possible dissociations between temporal and frontal MMN generators with age are required, especially in the context of pattern regularities.

### Regularity-Based Stream Segregation in Elderly Listeners

In the active-listening part of the current study, elderly listeners showed better foreground task performance when a task-irrelevant background stream carried a spectrotemporal regularity than when it did not. This is in line with the predictive-coding account of auditory stream segregation (Winkler et al., 2009, 2012). It is consistent with prior work on regularity-based stream segregation in young listeners (Andreou et al., 2011; Rimmele et al., 2012a), and informative with respect to previously conflicting results on whether this translates to elderly listeners: While de Kerangal et al. (2021) showed that the ability to track sources in an acoustic scene based on their regularities is largely preserved in elderly listeners, Rimmele et al. (2012a) had found an impairment of elderly listeners in regularity-based stream segregation. Since the current study closely followed the task and design of Rimmele et al. (2012a), we can now exclude task differences to underlie the different findings. The discrepancy of the current results with those of Rimmele et al. (2012a) is most likely due to issues of statistical power, with only 16 elderly listeners taking part in the previous study, compared to 30 elderly listeners in the current one. Alternatively, a procedural difference lies in the more comprehensive task training in the current study, from which the elderly may have benefitted. *Post hoc* analyses showed that the size of the behavioral benefit from regularity-based stream segregation in the two studies was not significantly different (though numerically higher in the current study), and that the behavioral benefit was significant when jointly analyzing both datasets. This underlines the necessity of replication studies with sufficient statistical power (Maxwell et al., 2015).

The fact that regularity-based stream segregation (i.e., behavioral benefit) does not correlate with peripheral hearing status (Figure 5B) nor with speech-in-noise comprehension (Figure 5C) is consistent with the findings from de Kerangal et al. (2021) who likewise found no such relations. This similar pattern of results in both studies is important on a theoretical level because it implies that the investigated regularity-based processing of complex acoustic scenes cannot trivially be explained by physical confounds in the stimulus setup (which might, e.g., put listeners with better peripheral hearing at an advantage). Instead, regularity-based processing of complex acoustic scenes appears to capture an independent ability, which is important to further investigate. The predictive-coding framework (Friston, 2005; Friston and Kiebel, 2009; Kanai et al., 2015; Denham and Winkler, 2017; Heilbron and Chait, 2018) provides an important theoretical basis for characterizing this ability, and in turn for developing a full understanding of auditory scene analysis and mitigating possible deficits thereof.

We conclude that elderly listeners *can* benefit from regularity-based stream segregation, but it is not known yet whether they can benefit *to the same extent* as young listeners. Answering the latter question would require taking measurements from a group of young listeners for comparison, which—as discussed for pattern regularity extraction above—was not in the focus of the current study. In view of the modified conclusion about the general ability of elderly listeners to perform regularity-based stream segregation, it seems warranted to verify the age group difference in this ability reported by Rimmele et al. (2012a) in an independent replication study. Similar to the ability for pattern regularity extraction, the ability for regularity-based stream segregation did not correlate with chronological age (Figures 5D, 6). Again, this does not rule out the existence of an age effect when comparing groups with a wider age range, as one would expect chronological age to dominate the results more when spanning a wider range. In any case, the observed strong inter-individual differences in the capacity to benefit from background regularity (both in the current and in the previous study) warrant further examination.

### Relations Between Pattern Regularity Extraction and Regularity-Based Stream Segregation

The key hypothesis of the current study was that listeners whose auditory system detects deviants more readily (as evidenced by MMN during passive listening) can also use predictability more easily for segregating sound sources from one another (as evidenced by regularity benefits in the active-listening task). This was expected to show up as a correlation between MMN amplitude and behavioral benefit. However, no such correlation was observed (Figure 5A). Since the inter-individual variation in both measures was high (see Figure 5A), the failure to find a correlation cannot be explained by floor or ceiling effects, or lack of variance. We must consider the possibility that part of the variation in the single-participant values reflects measurement error rather than true differences in the underlying ability. However, regarding MMN amplitudes, excessive measurement error seems unlikely given the significant correlation of individual mastoid MMN with age. Regarding behavioral benefit, robustness of the measurement was quantified by a Cronbach’s alpha of 0.58, which is considerably higher than the observed correlation of Δd’ benefit with any other measured variable.

To put potential concerns about measurement error further into perspective, it is important to note that well-established findings on auditory aging were replicated in the current study, even within the narrow age range (spanning 15 years) relative to studies comparing participants across different age groups (young vs. elderly). Specifically, we found that higher chronological age was accompanied by a decline in peripheral hearing ability (Figure 2A), which is consistent with prior work (e.g., Gates and Mills, 2005; Glyde et al., 2013). Increasing hearing thresholds challenge locating, detecting, discriminating, and comprehending sounds especially in complex acoustic environments, resulting in impaired speech comprehension (for a review see Pichora-Fuller and Souza, 2003; Martin and Jerger, 2005). In accordance with this, we confirmed that higher chronological age is accompanied by worse speech-in-noise comprehension (Figure 2B). The relation between peripheral hearing status and speech-in-noise comprehension, though significant, is far from being deterministic (Figure 2C). This underlines the partial independence of peripheral and central auditory processes (Plomp, 1978; Pichora-Fuller and Souza, 2003; Alain et al., 2006; Peelle and Wingfield, 2016). In any case, replication of these established findings in the current study rules out the possibility that severe measurement error masked inter-individual differences of relevant auditory abilities.

Other factors may help to understand the absence of a significant correlation between MMN amplitude and behavioral benefit. Before drawing premature conclusions on a putative dissociation between regularity extraction and regularity-based stream segregation, we should consider whether the two variables are valid indicators of the processes they are assumed to reflect. MMN is an indirect measure of regularity extraction (Schröger, 2005), though a dissociation between deviance detection and regularity extraction is unlikely under all current theoretical and modeling frameworks (Näätänen et al., 2007; Garrido et al., 2009; Fishman, 2014; Fitzgerald and Todd, 2020). A more imminent question is whether MMN *amplitude* gives a valid assessment of the strength or probability of deviance detection, or whether other processes may confound this measurement. Specifically, since we measured regularity extraction via MMN in a separate, passive-listening condition, MMN amplitude differences between participants may reflect different levels of attention to the sound stream while watching the silent documentary. Though attention effects on the MMN itself are small (Sussman, 2007), overlapping components such as the N2b (Novak et al., 1990) may confound the measurement and thereby artificially enhance the measured MMN amplitude. In fact, those participants who do not successfully disregard the irrelevant sound stream while focusing their attention on the video, might actually be those who also have trouble disregarding the task-irrelevant low-low-mid sound stream while focusing their attention on the task-relevant (A) stream. It may be the case that their negativity in the MMN latency range is counter-intuitively larger than those of many others because they fail to ignore the sound stream. This would be consistent with findings from cognitive aging in a wider sense, showing that the ability to inhibit task-irrelevant stimuli decreases with age across modalities and task types (e.g., Weeks and Hasher, 2018; Gaál et al., 2020). Interindividual differences in the ability to ignore task-irrelevant information (“resist interference”) would be one explanation why no consistent relation between MMN amplitude and benefit was found at group level. Future studies should mitigate this concern by measuring regularity extraction and deviant detection ability while participants’ auditory attention is more strongly controlled. It would also be advantageous to measure both abilities with the presence of the “A” sound stream.

Similarly, it could be questioned whether the behavioral benefit is a valid indicator of regularity-based stream segregation in every individual case. The underlying assumption is that more success in segregating the streams automatically translates into higher task performance. Yet this neglects the possibility that some participants may have trouble focusing their attention on the correct stream even when they succeed in segregating the streams (Gaeta et al., 2001; Healey et al., 2008; Horváth et al., 2009; Passow et al., 2012). Denham and Winkler (2017) summarize similarly conflicting results in their review article, stemming from the fact “that predictable sequences attract attention while also being easier to suppress.” They go on to conclude that “the presence of both tendencies may allow the influence of predictability to be easily modulated according to intrinsic preferences, attentional set and task demands” (Denham and Winkler, 2017). This consideration addresses contradictory findings across different studies, but it is well conceivable that it also applies within one study: In the current case, intrinsic preferences of individual participants may have obscured a systematic relation at group level. Therefore, an independent measurement of participants’ ability to focus on a given stream, and also to perform the task without the existence of a background stream, is needed to yield further insights into the involved processes. This would also tap more into related cognitive processes such as resistance toward interference, going beyond the very general cognitive screening in terms of verbal intelligence in the current study.

## Conclusion

In conclusion, due to a lack of an association between MMN amplitude and behavioral benefit we cannot provide support for the theoretical notion that extracting predictability and using predictability for decomposing acoustic mixtures are closely related processes. However, our observations do not rule out the possibility that such a relation may exist. Further studies are needed to rule out alternative explanations and to characterize the involved processes in more detail. The fact that elderly participants are successful in auditory regularity extraction and regularity-based stream segregation at group level, and that substantial inter-individual variation can be captured in these abilities with the present paradigm, provides a promising basis for further explorations into the involved processes.

## Data Availability Statement

The raw data supporting the conclusions of this article will be made available by the authors, without undue reservation.

## Ethics Statement

The studies involving human participants were reviewed and approved by the Ethics Committee of the University of Oldenburg. The participants provided their written informed consent to participate in this study.

## Author Contributions

AB, AF, and SD contributed to conception and design of the study. AF collected the data. CN and AF analyzed the data. AB and SD reviewed the data analysis. CN and AB wrote the first draft of the manuscript. All authors contributed to manuscript revision, read, and approved the submitted version.

## Conflict of Interest

The authors declare that the research was conducted in the absence of any commercial or financial relationships that could be construed as a potential conflict of interest.

## Publisher’s Note

All claims expressed in this article are solely those of the authors and do not necessarily represent those of their affiliated organizations, or those of the publisher, the editors and the reviewers. Any product that may be evaluated in this article, or claim that may be made by its manufacturer, is not guaranteed or endorsed by the publisher.

## Footnotes

1
https://www.hoertech.de/en/devices/olsa.html

2 Note that this assignment is to some degree arbitrary since the blocks were not administered in a paired manner.

3 We verified in a *post hoc* analysis that this listener’s data (with its inconsistent estimates of the Δd’ effect) did not affect the other analyses in any way. None of the correlations reported in the manuscript changed qualitatively when excluding this listener’s data.
